# 

**Published:** 1891-03

**Authors:** 


Vol. XII.] PUBLISHED MONTHLY AT THREE DOLLARS A YEAR. SINGLE COPIES, THIRTY CENTS. [No. 3.

### THE JOURNAL OF COMPARATIVE MEDICINE AND VETERINARY ARCHIVES.

EDITED BY

W. A. CONKLIN, Ph. D., D. V. S.,

*Director of Zoological Gardens, New York.*

RUSH SHIPPEN HUIDEKOPER, M. D., Veterinarian, (*Alfort.*)

*Professor of Sanitary Medicine and Veterinary Jurisprudence, American Veterinary College, New York.*

MARCH, 1891.

WILLIAM R. JENKINS, Publisher,

New York.

LONDON: BALLIERE, TINDALL & COX.

